# Hematological parameters of sheep and goats fed diets containing various amounts of water hyacinth (*Eichhornia crassipes*)

**DOI:** 10.3389/fvets.2024.1286563

**Published:** 2024-03-28

**Authors:** Yared Fanta, Yisehak Kechero, Nebiyu Yemane

**Affiliations:** ^1^Department of Animal and Range Sciences, Wolaita Soddo University, Wolaita Sodo, Ethiopia; ^2^Department of Animal Sciences, Arba Minch University, Arba Minch, Ethiopia

**Keywords:** hematological indices, goat, nutrition, sheep, water hyacinth

## Abstract

A major global barrier to increased animal output is nutrition. The use of aquatic plants, which were previously considered to be waste and needed a lot of labor to eliminate, has recently come to light due to the lack of feed during the dry season in the majority of tropical regions of Africa. The objectives of this study were therefore to see how different dietary *Eichhornia crassipise* inclusion rates affect the hematological indicators of Ethiopian Doyogena sheep and Woyto-Guji goats. Blood samples were taken from the jugular veins of 12 Doyogena sheep and 12 Woyto-Guji goats in a 2 × 4 randomized crossover design with two animal species, four diets, and four random periods (15 - day adaption period followed by a 7- day experimental diet in each period). The dietary inclusion rates *E. crassipise* were 0, 25, 50, and 75% that was used as a replacement for commercial concentrate mix diet in the treatment groups. The data was analyzed using the SAS software programme PROC GLM, and Pearson's correlation coefficient was calculated between hematological markers. The hemoglobin (Hb), red blood cell count (RBC), packed cell volume (PCV), mean corpuscular volume (MCH), and mean corpuscular hemoglobin (MCHC) results revealed substantial, RDW-SD, and WBC differences between animal species (*P* < 0.001). Sheep had greater WBC, Hb, RBC, PCV, RDW-SD, and RDW-CV levels, while goats had higher MCH and MCHC values (*P* < 0.001). For the analyzed hematological measures, the Pearson's correlation coefficient ranged from low to strong in terms of positive and negative associations (*P* < 0.05). Since all hematological indicators were closer to those of clinically healthy native Ethiopian sheep and goat breeds, feeding water hyacinth to sheep and goats up to a 75% inclusion level in diets without producing sickness may provide a remedy for adverse feed shortages.

## 1 Introduction

Small ruminants, such as sheep and goats, have significant importance in the livelihoods of many people in tropical regions, especially those who rely on native breed types. They contribute to income generation, food security, sustainable agriculture, and the empowerment of marginalized groups (1, 2). Doyogena Sheep and Woyto-Guji goat breeds are two potential indigenous livestock species in Ethiopia that play a vital role in the livelihoods and food security of Southern Ethiopian rural communities (3, 4). Despite its socioeconomic importance, small ruminant productivity is hampered by a number of complex and interconnected factors, such as poor genetics, poor reproductive performance, poor quality and varying seasonal availability of feed, high disease incidence and parasite challenges, and limited access to services and inputs (5). Among these, the seasonal shortage of feed was identified as one of the primary challenges in Ethiopia. This shortage refers to both the limited quantity and quality of available feed resources for livestock (6). Farm lands are used for crops during the rainy seasons, and the feed shortage persists even during those times (7).

Even though, agro-industrial by-products and commercial concentrate feeds are skyrocketing and thus restricting possibilities for resource-poor families. Under such circumstances, it becomes imperative for farmers to explore alternate, economically viable, and environmentally sound non-conventional feed resources like water hyacinth (*Eichhornia crassipes*), which could help alleviate feed shortages and improve the nutritional status of these animals. Water hyacinth is one of the noxious weeds that thrives and procreates in aquatic settings (8). It seriously jeopardizes the global environment, public health, the development of society, and access to clean water (9). Fresh plant matter may contain up to 95% water, which makes it challenging for harvesting and processing (10); transport, and post-harvest handling (11). Similarly, freshly harvested *E. crassipes* contains abrasive calcium oxalate crystals hurt the mouth and are which contribute to low palatability of various animal species (12). However, properly dried *E. crassipes* possesses potential as a feed resource for livestock due to its high nutritional value, such as proteins, carbohydrates, lipids, and so on Sotolu and Sule (13). Even in Ethiopia, study by Mekuriaw et al. (14) showed that wilted *E. crassipes* leave can effectively substitute concentrate mix up to 75% and promoting ideal growth of Washera sheep without notably toxicity. Considering its potential as an ingredient in livestock feed, there is a lack of comprehensive research on the influence of *E. crassipes* diets on the hematological indices of small ruminants such as sheep and goats. Hematological markers, particularly those linked to blood composition and immune response, provide significant information on the health and physiological status of farm animals (15). As a result, the purpose of this study was to look into the hematological indices of two sheep and goat breeds, Doyogena sheep and Woito-Guji goats that were fed varied amounts of water hyacinth in commercial concentrate mix and hay-based diets.

## 2 Materials and methods

### 2.1 About the study area

The experiment was conducted between October 2021 and February 2022 at the Arba Minch University livestock Research Farm at the Kulfo campus in Southern Ethiopia. The farm is located at a latitude of 6°2′21^′′^N and longitude 37°34′24^′′^E, and 435 km south of Addis Ababa, Ethiopia. It lies at an altitude of 1285 m above sea level, its average temperature is 29°c and average annual rainfall is 892 mm.

### 2.2 Animal management

Twelve intact yearling sheep lambs and 12 intact yearling goat bucks, Ethiopian Doyogena sheep and Woito-Guju goat breeds with average weights of 20.78 kg and 19.23 kg, respectively, were employed in an 84-day feeding trial and a 7-day digesting trial. Rams and bucks were quarantined and acclimatized for 4 weeks before onset of the experiment, during which they received a broad-spectrum anthelmintic (albendazole) against internal parasites, were sprayed with an accaricide (diazzinole) against external parasites, and were vaccinated against anthrax and pasteurelosis. During the acclimatization period, the animals were given time to adjust to their new environment and introduced to the experimental diets in order to adapt their rumen microbes. During the period, animals were weighed after overnight fasting on 2 consecutive days, and the average was taken as the initial body weight, ear tagged, and placed in separate metabolic pens. Each experimental animal was randomly assigned to treatment groups, and feed was provided in equal portions twice daily, with clean water and salt licks provided *ad libitum*.

### 2.3 Experimental design and treatments

The study employed a randomized two-by-four crossover design with two species and four treatment groups comprising three sheep and three goats in each period. Rams and bucks were given the experimental diets for 21 days for each phase, with blood samples collected on the last day. Animal weights were measured separately after an overnight fast to ensure consistency. The diet provision for the next period was adjusted based on body weight. The daily experimental dietary treatment (basal + supplement) fed to sheep and goats was fed at 3% of body weight, well-adjusted to provide minimum 8.36 MJ/kg metabolizable energy and 70 g DM CP/kg on a DM basis (16). The commercial concentrate, feed formulation that contained 33% maize, 10% nuge cake, 15% wheat middling, 40% wheat bran, 1% mineral premixes, and 1% salt; was purchased from Muza livestock feed maker and distributor, a private enterprise in Arba Minch. Grass hay was prepared from plant harvests from the Kulfo campus that were made from various plant species mixes. It was made up of plant groups such as *Asteraceae, Fabaceae, Poaceae, Commelinaceae*, and others, with *Fabacea* and *Asteraceae* dominating ones. A list of treatment groupings can be seen in Table 1.

**Table 1:** List of experimental dietary treatment.

**Treatments**	**Basal diet**	**Test diet**
T1	Grass hay	*E. crassipes* 0% + 100% commercial concentrate
T2	Grass hay	*E. crassipes* 25% + 75 % commercial concentrate
T3	Grass hay	*E. crassipes* 50% + 50 % commercial concentrate
T4	Grass hay	*E. crassipes* 75% + 25 % commercial concentrate

### 2.4 Blood sampling and collection

Hematological index analyses were carried out at the laboratory of the Nech Sar campus, Arba Minch University, Ethiopia. At the end of each experimental period, sheep and goats were restrained for blood collection. In order to perform hematological index analysis, 5 ml of blood samples were collected via the jugular vein into sterile test tubes containing the anti-coagulant Ethylenediaminetetraacetic acid (EDTA). Laboratory analyzed parameters included packed cell volume (PCV), white blood cell (WBC), hemoglobin (Hb), red blood cell (RBC), RBC indices such as mean corpuscular volume (MCV), mean corpuscular hemoglobin (MCH), and mean corpuscular hemoglobin concentration (MCHC), red blood cell distribution width standard deviation (RDW-SD), and red blood cell distribution width coefficient of variation (RDW-CV). This was done by using an automated hematology analyzer complete blood count machine (17).

### 2.5 Laboratory analysis of feed ingredients

The chemical composition of the feed samples used in feeding was analyzed at the chemistry laboratory of Arba Minch University. To determine the dry matter (DM), ash, Ether extract (EE) and Kjeldahl N following the procedures outlined in AOAC (18). The Crude Protein (CP) was determined by multiplying %N by 6.25, and the organic matter (OM) was estimated by subtracting the ash content from 100. The neutral detergent fiber (NDF), acid detergent fiber (ADF), and acid detergent lignin (ADL) were determined according to the procedure of Van Soest et al. (19).

### 2.6 Statistical analysis

Variance analysis was conducted following a 2 × 4 factorial layout in a randomized cross-over design using mixed model procedures (PROC MIXED) of SAS 2013 version 9.4. All hematological indices were firstly tested for normality and homoscedasticity with the Shapiro–Wilk's and Levene's test, respectively. Significant differences were declared at *P* < 0.05 and highly significant for *P* < 0.001. Means for hematological parameters were separated via the Tukey's HSD method and the level of significance was determined at *P* < 0.05. The Pearson correlation coefficient was used to evaluate the intensity of the association between hematological indices. The appropriate statistical model.


$$\begin{array}{l} {\text{Y}}_{\text{ijkl}}=\mu +\alpha_{\text{i}}+\beta_{\text{j}}+\gamma_{\text{k}}+\alpha \beta_{\text{ik}}+\Sigma_{\text{ijk}} \end{array}$$

Where,

Y_ijk_ = the response due to the animal i, in period j, treatment k, and interaction effects, μ = the overall mean effect, α_i_ = the fixed effect of the i^th^ species groups (I = sheep or goat) (subject; i =1, 2, 3…12), β_j_ = the random effect of the j^th^ collection period (j = 1, 2, 3, 4), γ_k_ = the fixed effect of the k^th^ treatment (k =1, 2, 3, 4), αβ_ik_ = the interaction effect between species i and treatment k, and Σ_ijkl_ = the random error.

## 3 Results

### 3.1 Chemical make-up of experimental feed ingredients

Table 2 displays the chemical composition analyses of the experimental feedstuffs used in the dietary interventions. Hay, a commercial concentrate combination, and water hyacinth all had dry matter values of 92.23%, 90.17%, and 92.34%, respectively. Ether extract ranged from 3.78% (hay) to 7.9% (concentration mix) while organic matter ranged from 89.12 (water hyacinth) to 93.15% (concentrate mix). When compared to grass-based hay, concentrate mix (CP, 17.98%) and water hyacinth leaves (CP, 12.05%) had higher crude protein levels. As anticipated, water hyacinth leaf content (5.72%) was twice that of hay. The most ash was found in water hyacinth (10.88%), while the least was in concentrate mix (6.85%). The concentrations of neutral detergent fibers, acid detergent fibers, and acid detergent lignin were highest in hay (66.29%, 42.18%, and 11.57%, respectively), and lowest in commercial concentrate mixture (38.47%, 27.92%, and 6.89).

**Table 2:** Chemical composition of experimental feedstuffs (% DM).

**Diets**	**DM (%)**	**OM (%)**	**Ash (%)**	**EE (%)**	**CP (%)**	**NDF (%)**	**ADF (%)**	**ADL (%)**
Hay	92.23	91.06	8.94	3.78	5.72	66.29	48.18	11.57
Concentrate	90.17	93.15	6.85	7.9	17.98	38.47	27.92	6.89
Water hyacinth	92.34	89.12	10.88	5.42	12.05	52.38	39.56	7.23

Source: Arba Minch University, Analytical Chemistry Laboratory Result.

### 3.2 Hematological profiles

The hematological profiles of sheep and goat breeds fed varying levels of water hyacinth (E. Crassipes) are presented in Table 3. The results revealed a significant variation in hematological parameters among the two species. The differences were found to be statistically significant (*P* < 0.001) for hemoglobin (g/dl), red blood cells (10^6^ /μL), packed cell volume (%), mean corpuscular hemoglobin (pg), and mean corpuscular hemoglobin concentration (g/L). Likewise, the white blood cell count (10^9^ /L) showed significant variation (*P* < 0.005) between the two species. Furthermore, the red blood cell distribution width standard deviation values varied significantly (*P* < 0.001) among the animals. Sheep displayed higher values for WBC count, Hb count, RBC count, PCV, RDW-SD, and RDW-CV, while goats had higher values for MCH and MCHC. There were no significant differences in mean corpuscular volume (fL) or red blood cell distribution width coefficient of variation. It also, appears that there were no significant differences observed among the treatment groups and no significant interactions between species and treatment being studied for any of the hematological parameters.

**Table 3:** Least square means for hematological parameters compared between sheep and goat fed graded levels of water hyacinth (*E. crassipes*).

**Parameters**	**Species**	**Treatment, Mean**					***P-*** **value**		
		**T1**	**T2**	**T3**	**T4**	**SEM**	**S**	**T**	**S × T**
WBC (10^9^/L)	Sheep	27.04 ^a^	24.76 ^a^	21.44 ^a^	25.11 ^a^	1.20	0.0041	0.083	0.083
	Goat	16.74 ^b^	19.22 ^b^	17.01 ^b^	17.58 ^b^				
Hb (g/dl)	Sheep	9.63 ^a^	10.58 ^a^	9.68 ^a^	8.96 ^a^	0.19	< 0.001	0.050	0.041
	Goat	8.72 ^b^	8.83 ^b^	7.77 ^b^	7.84 ^a^				
RBC (10^6^/μL)	Sheep	2.66 ^a^	2.82 ^a^	2.72 ^a^	2.49 ^a^	0.13	< 0.001	0.523	0.095
	Goat	0.60 ^b^	0.82 ^b^	0.47 ^b^	0.47 ^b^				
PCV (%)	Sheep	10.46 ^a^	11.18 ^a^	10.66 ^a^	9.57 ^a^	0.49	< 0.001	0.037	0.04
	Goat	2.36 ^b^	2.53 ^b^	1.78 ^b^	1.73 ^b^				
MCV (fL)	Sheep	41.77	42.17	41.28	40.38	0.76	0.868	0.082	0.098
	Goat	42.20	40.62	41.64	40.08				
MCH (pg)	Sheep	41.96 ^b^	45.52 ^b^	44.45 ^b^	41.10 ^b^	11.92	< 0.001	0.825	0.078
	Goat	186.28 ^a^	187.82 ^a^	206.72 ^a^	230.17 ^a^				
MCHC (g/L)	Sheep	97.92 ^b^	104.30 ^b^	105.67 ^b^	98.62 ^b^	25.79	< 0.001	0.074	0.04
	Goat	492.83 ^a^	447.13 ^a^	481.16 ^a^	555.07 ^a^				
RDW-CV (%)	Sheep	15.89	16.08	15.15	14.86	0.40	0.083	0.093	0.093
	Goat	14.37	13.84	14.05	14.03				
RDW-SD	Sheep	18.91 ^a^	19.35 ^a^	18.81 ^a^	18.59 ^a^	0.48	0.001	0.098	0.099
	Goat	15.57 ^b^	15.76 ^b^	15.36 ^b^	15.58 ^b^				

^a, b^ Means with different superscripts in the same column are significantly different (P < 0.05); S, species; T, treatment; S × T, species treatment interaction; WBC, white blood cells; Hb, hemoglobin; RBC, red blood cells, mean corpuscular hemoglobin concentration; PVC, packed cell volume; (RDW_CV), red cell distribution width coefficient of variation.

Tables 4, 5 summarize the results of the correlation study between hematological markers in sheep and goats, respectively. White blood cell count (WBC) and red blood cell count (RBC) (*r* = 0.46), as well as WBC and packed cell volume (PCV) (*r* = 0.39), were discovered to have positive associations in sheep. WBC and mean corpuscular volume (MCV) (*r* = −0.5), mean corpuscular hemoglobin (MCH) (*r* = −0.43), mean corpuscular hemoglobin concentration (MCHC) (*r* = −0.3), red cell distribution width coefficient of variation (RDW_CV) (*r* = −0.36), and red cell distribution width standard deviation (RDW_SD) (*r* = −0.38) were found to have negative correlations. Hb also had favorable relationships with MCV (*r* = 0.25), MCH (*r* = 0.55), and MCHC (*r* = 0.63). Furthermore, RBC were positively correlated with PCV (*r* = 0.95) but negatively correlated with MCV (*r* = −0.75), MCH (*r* = −0.85), MCHC (*r* = −0.78), RDW_CV (*r* = −0.33), and RDW_SD (*r* = −0.31). PCV also exhibited negative correlations with MCV (*r* = −0.51), MCH (*r* = −0.74), MCHC (*r* = −0.76), RDW_CV (*r* = −0.35), and RDW_SD (*r* = −0.35). On the contrary, MCV showed positive correlations with MCH (*r* = 0.80), MCHC (*r* = 0.57), and RDW_CV (*r* = 0.25), while MCH was positively correlated with MCHC (*r* = 0.94). Additionally, RDW_SD was positively correlated with MCHC (*r* = 0.14) and RDW_CV (*r* = 0.96).

**Table 4:** Pearson's correlation relationship between hematological parameters of Doyogena sheep fed varying incorporation rates of water hyacinth.

	**WBC**	**HGB**	**RBC**	**PCV**	**MCV**	**MCH**	**MCHC**	**RDW_CV**
HGB	0.029							
RBC	0.46^**^	−0.13						
PCV	0.39^**^	−0.07	0.95^***^					
MCV	−0.50^***^	0.25^*^	−0.75^***^	−0.51^***^				
MCH	−0.43^**^	0.55^***^	−0.85^***^	−0.74^***^	0.80^***^			
MCHC	−0.30^*^	0.63^***^	−0.78^***^	−0.76^***^	0.57^***^	0.94^***^		
RDW_CV	−0.36^*^	−0.09	−0.33^*^	−0.35^*^	0.25^*^	0.17	0.14	
RDW_SD	−0.38^**^	−0.09	−0.31^*^	−0.35^*^	0.2	0.14	0.14^***^	0.96^***^

^*^P < 0.05, ^**^P < 0.001, ^***^P < 0.0001.

**Table 5:** Pearson's correlation coefficient among hematological parameters of Woyto-Guji goat breed fed different inclusion rate of water hyacinth.

	**WBC**	**HGB**	**RBC**	**PCV**	**MCV**	**MCH**	**MCHC**	**RDW_CV**
HGB	0.37^*^							
RBC	0.34^*^	0.27^*^						
PCV	0.46^**^	0.58^***^	0.76^***^					
MCV	−0.43^**^	−0.01	−0.44^**^	−0.44^**^				
MCH	−0.40^**^	−0.07	−0.62^***^	−0.71^***^	0.78^***^			
MCHC	−0.29^*^	0.04	−0.52^***^	−0.60^***^	0.57^***^	0.88^***^		
RDW_CV	−0.27^*^	−0.02	−0.30^*^	−0.07	0.52^***^	0.16	0.02	
RDW_SD	−0.24^*^	−0.05	−0.16	−0.02	0.32^*^	0.02	−0.09	0.92^***^

^*^P < 0.05, ^**^P < 0.001, ^***^P < 0.0001.

In goats, positive correlations were observed between WBC and Hb (*r* = 0.37), RBC (*r* = 0.34), and PCV (*r* = 0.46). Negative correlations were found between WBC and MCV (*r* = −0.43), MCH (*r* = −0.40), MCHC (*r* = −0.29), RDW_CV (*r* = −0.27), and RDW_SD (*r* = −0.24). Hb showed positive correlations with RBC (*r* = 0.27) and MCH (*r* = 0.58), while RBC was positively correlated with PCV (*r* = 0.76). However, RBC exhibited negative correlations with MCV (*r* = −0.44), MCH (*r* = −0.62), MCHC (*r* = −0.52), and RDW_CV (*r* = −0.30). PCV also showed negative correlations with MCV (*r* = −0.44), MCH (*r* = −0.71), and MCHC (*r* = −0.60). On the other hand, MCV exhibited positive correlations with MCH (*r* = 0.78), MCHC (*r* = 0.57), RDW_CV (*r* = 0.52), and RDW_SD (*r* = 0.32), while MCH was positively correlated with MCHC (*r* = 0.88). Furthermore, RDW_SD was positively correlated with MCHC (*r* = 0.14) and RDW_CV (*r* = 0.92).

## 4 Discussion

### 4.1 Chemical composition of experimental diets

There is a wide range of values reported in different research for the crude protein (CP) content of grass-based hay in this study. Simone et al. (20) found a CP content of 4.60%, which is lower than ours. Kibret et al. (21) had the highest CP content at 9.53%, followed by Ayele et al. (22) at 7.9% and Yigzaw et al. (23) at 7.13%. Geleti et al. (24) and Teklehaymanot (25), for instance, reported CP levels of 5.8% and 5.56%, respectively, which are comparable to our findings. Variations in the CP content of grass hay among studies may be related to factors such as species composition, cultivation circumstances, and maturity stage at which the hay was harvested (26). The CP content of grass hay achieved in this investigation was < 7%CP required for microbial protein synthesis in the rumen to meet the basic maintenance demands of ruminants (19). The NDF, ADF, and ADL values in this study were greater than those published by Gulilat et al. (27), Woyessa et al. (28) and Mengistu et al. (29), which were 55.88%, 39.67%, and 6.4%, respectively. However, it had lower NDF, ADF, and ADL values when compared to Fikre et al. (30), which were 75.42%, 57.45%, and 14.42%, respectively. This inference validates the findings of Kibret et al. (21) and Teklehaymanot (25), who reported equivalent NDF, ADF, and ADL values of 65%, 42.6%, and 12.17%, respectively.

The CP concentration of the concentrate combination was comparable to the 19.2% reported by Ayele et al. (22) but less than the 23.40% and 24.54% reported by Simone et al. (20) and Abreha et al. (31), respectively. Amare and Girmay (32) reported a CP content of 16.3%, which is lower than our results. The variation in CP content of commercial concentrate mix among trials could be related to differences in the level and kind of components utilized in creating the concentrate combination, according to Simone et al. (20). When compared to previous research by Olafadehan and Okunade (33) and Abreha et al. (31), the concentrate mix in this study exhibited greater levels of NDF, ADF, and ADL. The corresponding figures were 30.6%, 14.2%, and 4.13%. Ayele et al. (22), Simone et al. (20), and Maurya et al. (34), on the other hand, reported identical NDF, ADF, and ADL values of 39.9%, 27.66%, and 6.23%, respectively.

The CP content of water hyacinth used in this study was comparable to the 11.9% reported by Patel et al. (35), but higher than the 10.3 to 10.4 and 10.5% reported by Hossain et al. (36) and Mako et al. (37), respectively, and lower than 14.4% reported by Mekuriaw et al. (14) and 18.03% CP reported by Aboud et al. (38). The water hyacinth NDF, ADF, and ADL values in this study were greater than those published by Banakar et al. (39), Mekuriaw et al. (14), and Tiwari et al. (40), which were 46.59%, 28.2%, and 5.35%, respectively. They were, however, lower than the NDF, ADF, and ADL values reported by Mako et al. (37) and Mako and Ikusika (41), which were respectively 65.9–77.9%, 36.5–39.7%, and 12.0%. Selim et al. (42) and Carneiro et al. (43), on the other hand, reported equivalent NDF, ADF, and ADL values of 54.8%, 29.8%, and 8.36%, respectively. The chemical makeup of water hyacinth may differ according on species, harvesting season, habitat and light, and water temperature, according to Shafy et al. (44), De Vasconcelos et al. (45), and Makkar et al. (46).

Lonsdale (47) classified feeds into low, medium, and high protein sources based on their protein composition. Feeds with < 12% CP were labeled as low protein sources, while those with 12 to 20% CP were labeled as medium protein sources. High protein sources were defined as feeds having a protein concentration >20% CP. Roughage feed can also be classed based on fiber fraction (NDF and ADF) value, which is crucial since it gives vital information on fiber content and animal digestibility. High-quality roughage feeds have NDF values below 45% and are easily digested, giving nutrients that are readily available. NDF-values in medium-quality roughage feeds range from 45% to 65%, striking a compromise between nutrient availability and fiber content. Low-quality roughage feeds have higher NDF-values and are more difficult to digest due to a higher proportion of indigestible components (48). Based on their ADF values, Kellems and Church (49) classified roughages similarly. Roughages with < 40% ADF were considered high quality, while those with more than 40% ADF were considered low quality. ADF denotes the less digestible components of the diet, such as cellulose and lignin. In this study, the CP content of grass hay was found to be low, indicating that the grass hay used in the study was of poor quality. The water hyacinth and concentrate mixture had a medium protein level.

### 4.2 Hematological profile

Generally, the hematological profile of animals plays a crucial role in understanding their overall health and wellbeing, such as their physiological status, diagnosis of diseases, monitoring treatment efficacy, and even assessing the impact of environmental factors on their health (50, 51). Moreover, it can also be a valuable tool in assessing feed quality, such as nutrient availability, mineral deficiencies, feed composition, digestibility and absorption in animals (52).

Perhaps, the most striking finding to emerge from this analysis was a substantial disparity in hematological parameters among sheep and goats. As expected, these differences were statistically significant, in terms of various blood parameters, including Hb, RBC, PCV, MCH, MCHC, WBC, and RDW_SD. These findings suggest distinct physiological variations between the species. These results are in accord with the observation by Etim et al. (53) that indicates significant variations in hematological parameters, including Hb, RBC, PCV, MCH, and MCHC, these variations are not limited to a specific population but are generalizable across different settings. Hematological indices of various livestock species are typically influenced by genetic and non-genetic factors (54). This distinct genetic variation between species may cause variations in their hematological profiles, influencing the production and functionality of red blood cells, hemoglobin, and other blood components. Hematological indices such as WBC count, Hb count, RBC count, PCV, RDW-SD, and RDW-CV values were higher in sheep. These results are consistent with those of Kiran et al. (55) findings, which showed, sheep consistently displayed higher levels of hematological values compared to goats.

Surprisingly, the study did not detect any significant effects of treatments or treatment-by-species interactions on hematological indices among sheep and goats. These findings align with several recent studies that used different dietary inclusion rates. For instance, WBC, RBC, Hb, PCV, MCV, MCH, and MCHC (56) values were not affected when sheep were fed varying dietary inclusions of alternative feedstuffs. Similarly, certain parameters such as PCV, Hb, WBC, and RBC (57) were not affected when sheep were fed varying dietary inclusions of Artemisia sieberi leaves. Moreover, WBC, RBC, MCH, MCHC, Hb, and PCV (58) values were not influenced by goats supplemented with Lablab purpureus and *Vigna unguiculata*. Furthermore, PCV, Hb, RBC, MCH, MCHC, and WBC (59) values were not affected by goat fed Mombasa or blue panic as a salt-tolerant alternative to replacing Alfalfa. PCV, Hb, RBC, MCV, MCH, and MCHC (60) values were not affected by West African Dwarf goats fed varying levels of treated sweet orange peels. The lack of significant effects observed in our study suggests that treatments and treatment-by-species interactions may not play a significant role in influencing hematological indices in the studied species. However, the findings of the current study do not support previous research by Kurtoglu et al. (61) and Osita et al. (62) on hematological indices influenced by diet.

### 4.3 White blood cell

The values for WBC in this study ranged between 21.44 and 27.04 × 10^9^/L for sheep and 16.74 and 19.22 × 10^9^/L for goats. The values obtained in this study are far higher than laboratory reference value by Jackson and Cockcroft (63) 4–12 × 10^9^ /L sheep and 4–13 × 10^9^/L goats. Comparable to the value range of 20.44 ± 1.02 × 10^9^/L and 27.0 ± 0.56 × 10^9^/L reported by Njidda et al. (64) for Yankasa and Ouda sheep, respectively, in the semi-arid region of Nigeria. In this study, an elevated WBC suggests an immunological reaction against contamination or toxic chemicals in the body, whereas a smaller count implies the presence of infectious infections or antigens in the body (65). The elevated levels of WBC observed in both species indicate that they have an immune system that is functioning properly.

### 4.4 Hemoglobin

Hemoglobin, an iron-rich protein, is found in red blood cells. It allows the blood to carry oxygen to all of the body's tissues (66). The Hb values were between 8.96 and 10.58 (g/dl) sheep and 7.77–8.83 (g/dl) goats. The values obtained in this study are almost in accord with laboratory reference value by Jackson and Cockcroft (63) 9–15 g/dl sheep and 8–12 g/dl goats. Moreover, this result is in line with the value range between 8.8 and 10.73 g/dl) reported by Dikko et al. (67) for Yankasa Sheep fed Brewers' Dried Grain, and 8.6 and 9.7 g/dl were reported by Pudjihastuti et al. (68) for domestic goats supplemented with urea palm sugar block. Generally, elevated concentrations of HB are attributed to a more pronounced capacity for overcoming infection with a disease, while lower levels are a sign of illness, infection, and inadequate nutrition (69, 70). The study indicates that, neither sheep nor goats suffer from microcytic hypochromic anemia, which is related to iron scarcity and scant utilization during the formation of Hb Olafadehan (71).

### 4.5 Red blood cell

The RBC value were between 2.49 and 2.82 (106/μL) sheep and 0.47–0.82 (106 /μL) goats and were not statistically different (P > 0.05) among the treatments. The values observed in this investigation are far below those observed by Jackson and Cockcroft (63) 9–15 × 10^6^ /μl sheep and 8–18 × 10^6^/μl goats. This abnormally low figure obtained in this study might be a clinical sign of eminent vulnerability to anemia-related disease conditions (72). Furthermore, the lowest levels of red blood cells may be a sign of anemia, internal bleeding, inadequate iron consumption, vitamin deficiency, or other health problems (64). However, comparison of the findings with those of other recent studies reported closer values of 3.40 ± 0.12, 3.41 ± 0.12, 3.11 ± 0.12, and 3.72 ± 0.12 for different sheep breeds reared by Agbaye et al. (73). Moreover, 2.39–3.51 × 10^6^/μl WAD sheep fed Ensiled maize stover and concentrate supplement had no clinical signs of disease (74).

### 4.6 Packed cell volume

PCV is a metric representation of the quantity of volume the red blood cells occupy in relation to the overall volume of blood in a sample of capillary, venous, or arterial blood (75). PCV values ranged from 9.57 to 11.18 % in sheep and 1.73–2.53 % in goats. The values observed in this study were far below the reference range by Jackson and Cockcroft (63) for sheep (27.0–45.0%) and goats (22.0–38.0%). The study indicates that the animal may be at risk for developing macrocytic anemia due to a vitamin B12 or folate deficiency (76).

### 4.7 RBC indices

RBC indices are metrics that are crucial to diagnosing the underlying cause of anemia (77). MCV, MCH and MCHC are considered RBC indices give details about average cell size, hemoglobin content, and hemoglobin concentration, respectively (78). The MCV values of sheep 40.38 - 42.17 (fL) obtained in this study were closer to the range value 45.5–46.78 (fL) obtained by Orzuna et al. (79) for sheep supplemented with a Polyherbal Mixture; little bit higher than the reference range by Jackson and Cockcroft (63) for sheep 28–40 (fL) and goats 16–25 (fL), but lower than 62.2 (fL) reported by Egbe-Nwiyi et al. (80) of apparently healthy goats. The MCV value in obtained this study implies that the animal is exposed to macrocytic anemia due to vitamin B12 or folate deficiency (76). Contrary to expectations, results pertaining to MCH in this study were by far higher than the laboratory reference for hematological parameters of sheep 8–12 (pg) and goats 5.2–8 (pg) Jackson and Cockcroft (63). An elevated MCH can signify the existence of reticulocytes or hemolysis, whereas a lower MCH is thought to correspond to a severe iron deficiency (78). Similarly, MCHC has been suggested to be the most accurate of the RBC indices, and it might rise when there is an instance of hemolysis or fall in the case of reticulocytosis and iron deficient anemia (78). MCHC values in this study far lower for sheep 310–340 (g/L) and far higher than goats 300–360 (g/L) than the laboratory reference for hematological parameters (63).

### 4.8 Red cell distribution width

RDW, an estimate derived from the red blood cell distribution curves obtained from automated hematology analyzers, was carried out to gauge the erythrocytes' size variability in circulation (81). RDW-CV levels in this study range between 14.86 and 16.08% in sheep and 13.84–14.37% in goats. These values are somewhat closer to Manchega Spanish sheep (16.44 ± 0.34%) Bórnez et al. (82) and Pramenka sheep (15.46%) in Livno fields, Ohran (83). However, it was lower than 24.16 ± 1.61 % that of Wonosobo sheep (84). On the other hand, RDW levels of goats obtained in this study were lower than those of Mohammed et al. (76) 28.75 ± 3.04% for the Damascus goat, and Reiten et al. (85), 23–35% for the Norwegian Dairy Goat Breed. Elevated concentration of RDW outside the typical reference range, prognostic for iron deficiency anemia, folic acid and/or vitamin B12 deficiencies, autoimmune or hemolytic anemia, myelodysplastic syndrome, or sickle cell disease (77).

### 4.9 Correlation coefficients between homatological indices

The Pearson correlation coefficient for hematological indices in this study exhibited low to high, positive and negative levels of associations. So far, the study has broadly aligned and deviated from recent studies working with a variety of experimental designs and sheep and goat breeds. Antunović et al. (86) reported a significant negative association between WBC and MCV (*r* = 0.22) for the Merinolandschaf sheep breed. Karki et al. (87) reported a significant positive association between RBC and HCT (*r* = 0.81) for the Katahdin sheep breed. A negative association between RBC and MCV (*r* = 0.29), RBC and MCH (*r* = 0.59), and RBC and MCHC (*r* = 0.42) was found by Karki et al. (87) for the Katahdin sheep breed and HCT and MCHC (*r* = 0.24) by Antunović et al. (86) for Merinolandschaf sheep. However, the findings of the current study deviate from the previous research. Fadare et al. (88) report a significant negative association between WBC and RBC (*r* = 0.72) and WBC and HTC (*r* = 0.41) for white-colored West African Dwarf sheep. Antunović et al. (86) reported significant negative correlations between Hb and MCV (*r* = 0.21); Hb and MCHC (*r* = 0.22); and MCH and MCHC (*r* = 0.63) for Merinolandschaf sheep.

The results pertaining to certain hematological indices of goats in this study support recent studies using different goat breeds with different experimental designs. A significant positive correlation obtained between Hb and RBC (*r* = 0.805) and PCV (*r* = 0.424) in goats (89); between Hb and HCT (*r* = 0.57) for beef (90); and between MCV and MCH (*r* = 0.95) Perumal et al. (91) and Karki et al. (87) findings reported that RBC was negatively correlated with MCV (*r* = −0.58) and MCH (*r* = −0.71) in the kiko goat breed. In contrast, the results of this investigation differ from those of the other studies. A significant negative association between WBC and RBC (*r* = 0.16) (92); and between WBC and RBC (*r* = 0.35) (93) for WAD goats whereas a significant positive correlation was observed between RBC and MCHC (*r* = 0.36) for kiko goat breeds (87).

## 5 Conclusion

In conclusion, the study successfully determined that different levels of water hyacinth inclusion in the diets of sheep and goats did not have any detrimental effects on their hematological parameters. There were a substantial disparity observed among the hematological parameters of sheep and goats, but these differences were not influenced by the treatments or treatment-species interactions. While certain parameters such as PCV, RDW, RBC, and its indices deviated from the normal range, none of the animals showed any clinical signs of conditions like haemolysis, microcytic hypochromic anemia, or inflammatory response to the treatment diets. Pearson correlation coefficient for hematological indices showed both positive and negative relationships, ranging from low to high. Overall, these findings suggest that dried water hyacinth meal can be used as a substitute for up to 75% of commercial concentrate diets without affecting the hematological indices of sheep and goats. Further validation of these findings necessitates additional studies. These future studies should delve deeper into evaluate the effects of prolonged water hyacinth inclusion in the diets of sheep and goats on their hematological profiles. Additionally, it would be valuable to investigate the potential accumulation of any toxins or contaminants from water hyacinth in the animals' systems and how it may impact their hematological health.

## Data availability statement

The datasets presented in this study can be found in online repositories. The names of the repository/repositories and accession number(s) can be found at: https://doi.org/10.5281/zenodo.8304949.

## Ethics statement

The Animal Research Ethics Committee of Arba Minch University approved the research methodologies and procedures, verifying that they follow ethical standards. The committee issued a certificate (ref. no AMU/AREC/4/2016, dated 20, September, 2021) stating that the study was approved and carried out in an ethical and responsible manner. The study was conducted in accordance with the local legislation and institutional requirements.

## Author contributions

YF: Conceptualization, Data curation, Formal analysis, Funding acquisition, Investigation, Methodology, Project administration, Resources, Software, Validation, Visualization, Writing – original draft, Writing – review & editing. YK: Supervision, Writing – review & editing. NY: Supervision, Writing – review & editing.
